# Five‐year results of a phase II trial of preoperative 5‐fluorouracil, epirubicin, cyclophosphamide followed by docetaxel with capecitabine (wTX) (with trastuzumab in HER2‐positive patients) for patients with stage II or III breast cancer

**DOI:** 10.1002/cam4.1472

**Published:** 2018-03-26

**Authors:** Frankie Ann Holmes, Beth A. Hellerstedt, John E. Pippen, Svetislava J. Vukelja, Rufus P. Collea, Darren M. Kocs, Joanne L. Blum, Kristi J. McIntyre, Minal A. Barve, Barry D. Brooks, Cynthia R. Osborne, Yunfei Wang, Lina Asmar, Joyce O'Shaughnessy

**Affiliations:** ^1^ US Oncology Research McKesson Specialty Health The Woodlands Texas; ^2^ Texas Oncology‐Houston Memorial City Houston Texas; ^3^ Texas Oncology‐Austin Central Austin Texas; ^4^ Texas Oncology‐Baylor Sammons Cancer Center Dallas Texas; ^5^ Texas Oncology Tyler Texas; ^6^ New York Oncology Hematology Albany New York; ^7^ Texas Oncology Round Rock Texas; ^8^ Texas Oncology Dallas Presbyterian Hospital Dallas Texas; ^9^ Texas Oncology Medical City Dallas Dallas Texas

**Keywords:** Breast cancer, capecitabine, FEC, preoperative chemotherapy, trastuzumab

## Abstract

We aimed to increase pathologic complete response (pCR) in patients with invasive breast cancer by adding preoperative capecitabine to docetaxel following 5‐fluorouracil, epirubicin, cyclophosphamide (FEC) (with trastuzumab for patients with HER2‐positive disease) and to evaluate 5‐year disease‐free survival (DFS) associated with this preoperative regimen. Chemotherapy included four cycles of FEC100 (5‐fluorouracil 500 mg/m^2^, epirubicin 100 mg/m^2^, cyclophosphamide 500 mg/m^2^ IV on Day 1 every 21 days) followed by 4 21‐day cycles of docetaxel (35 mg/m^2^ days 1 and 8) concurrently with capecitabine (825 mg/m^2^ orally twice daily for 14 days followed by 7 days off) (wTX). For HER2‐positive patients, treatment was modified by decreasing epirubicin to 75 mg/m^2^ and adding trastuzumab (H) in standard doses (FEC75‐H →wTX‐H). The study objective was to achieve a pCR rate in the breast and axillary lymph nodes of 37% in patients with HER2‐negative breast cancer and of 67% in patients with HER2‐positive breast cancer treated with preoperative trastuzumab. A total of 186 patients were enrolled on study. In an intent‐to‐treat analysis, the pCR rate was 31% (37/118, 95% CI: 24–40%) in the HER2‐negative patients, 24% (15/62, 95% CI: 14–37%) in ER‐positive/HER2‐negative patients, 39% (22/56, 95% CI: 27–53%) in the ER‐negative/HER2‐negative patients, and 46% (29/63, 95% CI: 34–48%) in the HER2‐positive patients. The pCR rate in the 40 trastuzumab‐treated patients was 53% (21/40, 95% CI: 38–67%). Grade 3 and 4 adverse events included neutropenia, leukopenia, diarrhea, and hand‐foot skin reactions. One trastuzumab‐treated patient developed grade 3 cardiotoxicity, and 4 others experienced grade 1–2 decrements in left ventricular function; all five patients’ cardiac function returned to their baseline upon completion of trastuzumab. At 5 years, disease‐free survival was 70% in the HER2‐negative population (78% in ER‐positive/HER2‐negative and 62% in the ER‐negative/HER2‐negative patients) and 80% in the HER2‐positive patients (87% in the trastuzumab‐treated HER2‐positive patients). At 5 years, overall survival was 80% in the HER2‐negative population (88% in ER‐positive/HER2‐negative and 71% in the ER‐negative/HER2‐negative patients) and 86% in the HER2‐positive patients (94.5% in the trastuzumab‐treated HER2‐positive patients). FEC100 (FEC75 with trastuzumab) followed by weekly docetaxel plus capecitabine, with or without trastuzumab is a safe, effective preoperative cytotoxic regimen. However, the addition of capecitabine to docetaxel following FEC, with or without trastuzumab, did not increase pCR rates nor 5‐year DFS over the rates that have been reported with standard preoperative doxorubicin/cyclophosphamide (AC) followed by paclitaxel, with or without trastuzumab. Therefore, the use of capecitabine as part of preoperative chemotherapy is not recommended.

## Introduction

Preoperative treatment of breast cancer using four cycles of standard doxorubicin 60 mg/m^2^ with cyclophosphamide 600 mg/m^2^ (AC) IV every 3 weeks followed by docetaxel (T) 100 mg/m^2^ IV every 3 weeks for four cycles (AC→T), is associated with a 26% pCR in an unselected patients, and is an accepted standard of care regimen 1, 2. At the time this study was developed, achievement of pCR was interpreted as a marker for long‐term survival in all patient subsets whereas it is now believed that the prognostic utility of pCR differs according to the molecular subset 3, 4.

Treatment with an adjuvant anthracycline has been shown to improve disease‐free (DFS) and overall survival (OS) compared with non‐anthracycline regimens 1. The taxanes, paclitaxel and docetaxel, have been shown as well to significantly improve DFS and OS in early stage breast cancer 5. In the metastatic setting, O'Shaughnessy et al. 6 demonstrated an OS benefit with oral capecitabine, 1250 mg/m^2^ bid for 14 days followed by 7 days off, plus docetaxel 75 mg/m^2^ compared with docetaxel at 100 mg/m^2^ providing the rationale to evaluate combined taxane/capecitabine therapy in early breast cancer patients. In August 2002, O'Shaughnessy et al. 7 had begun just such a trial in 2611 pts who received doxorubicin 60 mg/m^2^ with cyclophosphamide 600 mg/m^2^ IV every 21 days (standard “AC”) followed by either docetaxel alone or the doublet with capecitabine as above. The present trial, begun in March 2004, aimed to test this strategy in the neoadjuvant chemotherapy setting, in which the rate of pCR might provide evidence of efficacy more rapidly.

In patients with HER2‐positive tumors, trastuzumab given concurrently with FEC75 and paclitaxel had been shown to substantially increase the pCR rate over that achieved with chemotherapy alone, without dose‐limiting toxicity 8, although the development of cardiac toxicity is of concern when anthracyclines and trastuzumab are co‐administered 9.

The objectives of this phase II study were to define the pCR and 5‐year DFS and OS rates associated with preoperative FEC followed by weekly docetaxel plus capecitabine, with or without trastuzumab, in patients with HER2‐negative or HER2‐positive breast cancer. We also sought to correlate the development of a pCR with baseline tumor gene‐expression profiles and these results have been previously reported 10, 11. We also correlated the probability of developing a pCR with the results of the ChemoFx chemotherapy sensitivity assay performed on tumor biopsies obtained prior to therapy initiation, and have published these results 12. Herein, we present the pCR rates and 5‐year DFS and OS results obtained following treatment with preoperative FEC followed by weekly docetaxel plus capecitabine, with or without trastuzumab, in patients with stage II or III breast cancer.

## Methods

### Study design and treatment plan

This phase II, open‐label study was approved by the US Oncology central institutional review board. All patients provided written informed consent before enrolling.

The primary objective was to determine whether the pCR rate could be increased with the addition of capecitabine. After Buzdar et al.'s practice‐changing report of preoperative trastuzumab in HER2‐positive patients in 2005 et al. showing a significant increase in pCR rates, from 25% to 67%, when trastuzumab was added to a preoperative regimen of weekly paclitaxel followed by FEC for patients with HER2‐positive breast cancer 8, the protocol was amended and the primary objective was quantified by subtype. In the HER2‐negative patient cohort, the objective was to determine whether preoperative therapy with FEC100 followed by wTX (doses provided below) would result in a pCR rate in the breast and axillary lymph nodes of 37%.

The primary objective in the HER2‐positive cohort was to determine whether preoperative trastuzumab, given concurrently with FEC75 followed by wTX would result in a breast and lymph node pCR rate of 67% as had been shown by Buzdar et al. 8 using FEC75 with trastuzumab.

The secondary objectives were to perform gene expression analysis to discern a gene expression profile that would predict pCR 13 and to determine the overall safety of the preoperative regimens, including the cardiac safety of the combination of concurrent trastuzumab and chemotherapy with FEC75 followed by wTX.

Inclusion criteria were: patients ≥18 years of age with American Joint Committee on Cancer (AJCC) 14 clinical stages IIA through IIIC invasive breast cancer, a core or open biopsy that confirmed invasive breast cancer, Eastern Cooperative Group (ECOG) 15 performance status 0–1, left ventricular ejection fraction (LVEF) ≥50%, able to tolerate eight cycles of preoperative chemotherapy including four cycles of an anthracycline, and willingness to undergo a mandatory research breast cancer biopsy to obtain tissue for gene expression profiling and for the ChemoFx assay.

### Treatment

HER2‐negative patients and HER2‐positive patients treated before 2005 received FEC100: 5‐fluorouracil 500 mg/m^2^, epirubicin 100 mg/m^2^, cyclophosphamide 500 mg/m^2^ IV on Day 1 of every 21‐day cycle for 4 cycles, followed by wTX: docetaxel 35 mg/m^2^ days 1 and 8 concurrently with capecitabine 825 mg/m^2^ orally twice daily for 14 days followed by 7 days off on a 21‐ day cycle for four cycles. The days 1 and 8 schedule of docetaxel was chosen after early toxicity results from our ongoing adjuvant trial of the same combination suggested that the gastrointestinal toxicity, especially diarrhea, on the combined docetaxel and capecitabine arm was increased when docetaxel and capecitabine followed initial therapy with AC 7. Additionally, it was hypothesized that the known beneficial effect of docetaxel to upregulate thymidine phosphorylase, essential for efficacy of capecitabine, might be enhanced with weekly administration of docetaxel while simultaneously decreasing the incidence of diarrhea 16. The total planned treatment period was 24 weeks.

**Figure 1:**
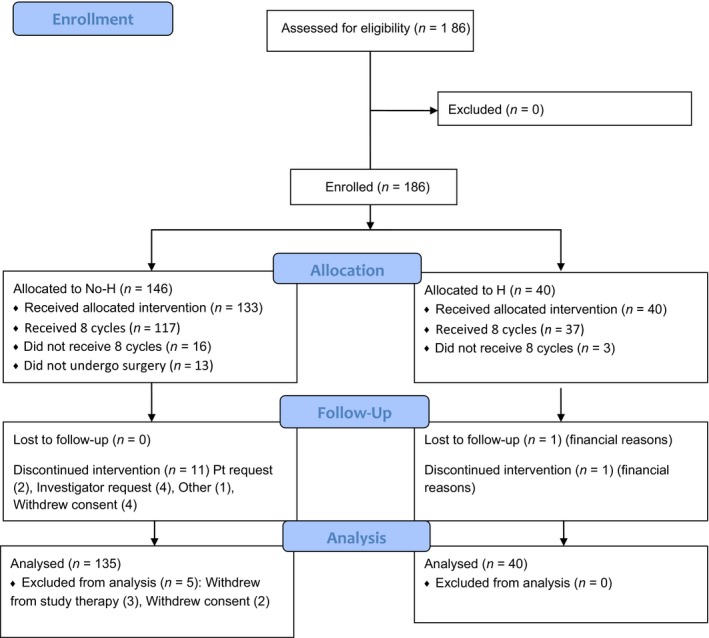
02‐103 CONSORT flow diagram.

After 2005, HER2‐positive patients received FEC75: the same regimen as FEC100 but epirubicin 75 mg/m^2^ as in Buzdar et al. to minimize cardiac toxicity with concurrent trastuzumab (Fig. 1). Trastuzumab was administered with an initial loading dose of 4 mg/kg IV on day 1 followed by 2 mg/kg IV weekly for the entire 24 weeks of treatment given concurrently with FEC75 followed by wTX.

After the completion of preoperative chemotherapy, patients underwent definitive surgical resection with lumpectomy or mastectomy as well as axillary dissection. Patients received locoregional radiation therapy as prescribed per the community standard of care. Patients with ER‐positive breast cancer were treated postoperatively with at least 5 years of endocrine therapy. Six additional months of adjuvant trastuzumab was administered at the discretion of the treating physician; after June 2005, all patients completed a total of 1 year of trastuzumab.

### On‐study assessments

Pretreatment evaluations included chest and abdominal computed tomography (CT) scans, radionuclide bone scan, or positron‐emission tomography (PET) scan. Subsequent imaging studies were performed as clinically indicated. Baseline cardiac function evaluation of left ventricular ejection fraction (LVEF) was assessed by multi‐gated acquisition scan angiography (MUGA) or echocardiography.

Patients underwent pretreatment biopsies for gene expression and ChemoFX assay testing. Results from analyses correlating pathologic response with gene expression profiles were conducted by our collaborators at U.T. M. D. Anderson and from in vitro chemotherapy sensitivity testing using the ChemoFX assay, have been previously published 10, 11, 12, 13.

Before each 21‐day treatment cycle, patients had a history and physical examination, complete blood count (CBC) with differential and platelet count and comprehensive metabolic profile (CMP). CBCs were obtained weekly during the preoperative chemotherapy. Definitive surgery was performed between days 22 and 29 of the last cycle of wTX.

All patients, including those who discontinued treatment because of disease progression, intolerable toxicity, treatment interruption of more than 2 weeks, or intercurrent illness as well as those who completed treatment, were followed for survival status every 6 months for 5 years.

### Statistical analysis

A total of 186 patients (118 HER2‐negative, 64 HER2‐positive and 4 HER2 unknown) were enrolled to determine whether preoperative therapy with four cycles of FEC100 or four cycles of FEC75, respectively, followed by wTX for four cycles, with or without trastuzumab, would result in a breast and lymph node pCR rate of 37% in HER2‐negative patients, and of 67% in HER2‐positive patients treated with trastuzumab, respectively. Using the 26% pCR rate observed in the NSABP B‐27 trial with AC followed by docetaxel as a reference standard, the study had 80% power with a significance level of 0.05 to detect a 37% pCR rate in the HER2‐negative population (1). The study was initiated before trastuzumab was approved for neoadjuvant use and thus was not powered to address the pCR in HER2‐positive patients, however, a benchmark pCR of 67% was chosen based on the initial report of Buzdar et al. 8. All treated patients, regardless of whether they underwent definitive surgery, were included in the analyses of pCR rates.

Descriptive statistics were used to characterize the patient population. pCR rates and clinical objective response rates were determined based on the intent‐to‐treat and evaluable populations and 2‐sided 95% confidence intervals were calculated. Response Evaluation Criteria in Solid Tumors (RECIST) 17 was used to determine clinical response to treatment. pCR was defined as no invasive disease in the resected breast tissue and axillary lymph nodes (ypT0/is ypN0). OS (the time from the date of treatment initiation to the date of death or date of last contact) and DFS (time from the date of treatment initiation to the date of disease recurrence, new primary breast cancer or death from any cause) were estimated using the Kaplan–Meier method 18. If the patient had not developed disease recurrence or new primary breast cancer and had not died, the patient was censored on the date of last follow‐up. Toxicity was evaluated in the safety population (all patients who received at least 1 dose of study drug). Common Terminology Criteria for Adverse Events (CTCAE) Version 3.0 19 was used to grade and report toxicities and AEs. SAS software (version 9.2, SAS Institute Inc, Cary, NC) was used to perform the analyses.

## Results

From March 2004 through April 2006, 186 eligible patients were enrolled in the study: 118 HER2‐negative patients (62 ER+ patients and 56 ER‐negative patients), and 64 HER2–positive patients (40 treated with trastuzumab and 24 treated without trastuzumab). HER2 status was unknown in four patients (Fig. 1). Median age of all patients was 49 years. Ninety percent of patients had invasive ductal cancer, 56% of which was grade 3. Ninety‐six (52%) patients had Stage II and 90 (48%) patients had Stage III disease. In the HER2‐negative cohort, 46% of the cancers were ER‐positive/progesterone receptor (PgR)‐positive, 7% were ER‐positive/PgR‐negative, 5% were ER‐negative/PgR‐positive, and 42% were ER‐negative/PgR‐negative. In the HER2‐positive cohort, 30% of the cancers were ER‐positive/PgR‐positive, 8% were ER‐positive/PgR‐negative, 3% were ER‐negative/PgR‐positive, and 59% were ER‐negative/PgR‐negative. Patient and tumor characteristics are summarized in Table 1. One patient withdrew consent to participate in the study and therefore 185 patients comprised the evaluable and safety population. Definitive surgery was performed in 173 patients.

**Table 1:** Demographics and baseline characteristics (ITT population)

	HER2 negative		HER2 positive		Total
	ER + *n* = 62 (%)	ER − *n* = 56 (%)	No Tras *n* = 24 (%)	Tras *n* = 40 (%)	*N* = 186 (%)
Age					
Median	50.4	50.8	49.8	48.8	49.9
Range	28.6–73.3	25.9–83.5	30.5–74.1	24.1–69.7	24.1–83.5
Race					
Caucasian	54 (87)	44 (79)	17 (71)	33 (83)	151 (81)
Black	6 (10)	9 (16)	2 (8)	5 (13)	23 (12)
Hispanic	1 (2)	3 (5)	2 (8)	2 (5)	8 (4)
Other	1 (2)	0	3 (13)	0	4 (2)
Histology					
Ductal	48 (77)	54 (96)	24 (100)	36 (90)	166 (89)
Lobular/mixed	14 (23)	0	0	2 (5)	16 (9)
NOS	0	2 (4)	0	2 (5)	4 (2)
Histologic Grade					
Grade 1	4 (7)	0	0	0	4 (2)
Grade 2	22 (36)	10 (18)	7 (29)	11 (28)	51 (27)
Grade 3/4	22 (36)	42 (75)	17 (71)	22 (55)	104 (56)
Unknown	14 (23)	4 (7)	0	7 (18)	27 (15)
Stage at diagnosis					
II	35 (57)	27 (48)	11 (46)	20 (50)	96 (52)
III	27 (44)	29 (52)	13 (54)	20 (50)	90 (48)
ER PR status					
ER+ PR+	54 (87)	0	8 (33)	11 (28)	73 (39)
ER+ PR−	8 (13)	0	1 (4)	4 (10)	14 (8)
ER− PR+	0	6 (11)	2 (8)	0	8 (4)
ER− PR−	0	50 (89)	13 (54)	25 (63)	90 (48)
Unknown	0	0	0	0	1 (1)

Note: Four additional patients were HER2 unknown and data were not shown in this table.

### Efficacy

Eight planned cycles of preoperative chemotherapy were completed by 95 (81%) and 36 (90%) patients in the HER2‐negative and trastuzumab‐treated HER2‐positive cohorts, respectively. Of the 118 patients with HER2‐negative disease in the intent‐to‐treat population, the pCR rate was 31% (37/118, 95% CI: 24–40%). The clinical complete response (cCR) rate in this population was 46% (54/118, 95% CI: 37–55%). In the 40 patients with HER2‐positive disease who received trastuzumab, all of whom underwent surgery, the pCR rate was 53% (21/40, 95% CI: 38–67%), and the cCR rate was 43% (17/40, 95% CI: 27–59%). In the 23 patients with HER2‐positive disease who did not receive trastuzumab, the pCR rate was 35% (8/23, 95% CI: 16–57%) (Table 2). Exploratory analyses of pCR rates by ER status in the HER2‐negative and trastuzumab‐treated HER2‐positive patients are shown in Table 2.

**Table 2:** Pathologic complete response rates by study arm—intent‐to‐treat population

Study arm	No. of patients	pCR rates breast and axilla *n* (%)	95% CI (%)
HER2‐negative/ER‐positive	62	15 (24.2)	(14.2, 36.7)
HER2‐negative/ER‐negative	56	22 (39.3)	(26.5, 53.2)
HER2‐positive/No Trastuzumab	23^a^	8 (34.8)	(16.4, 57.3)
HER2‐positive/Trastuzumab	40	21 (52.5)	(36.1, 68.5)
HER2 status unknown	4	2 (50.0)	(6.8, 93.2)

a One patient in the HER2‐positive/No Trastuzumab cohort did not receive any study treatment and was excluded from the efficacy analysis.

**Figure 2:**
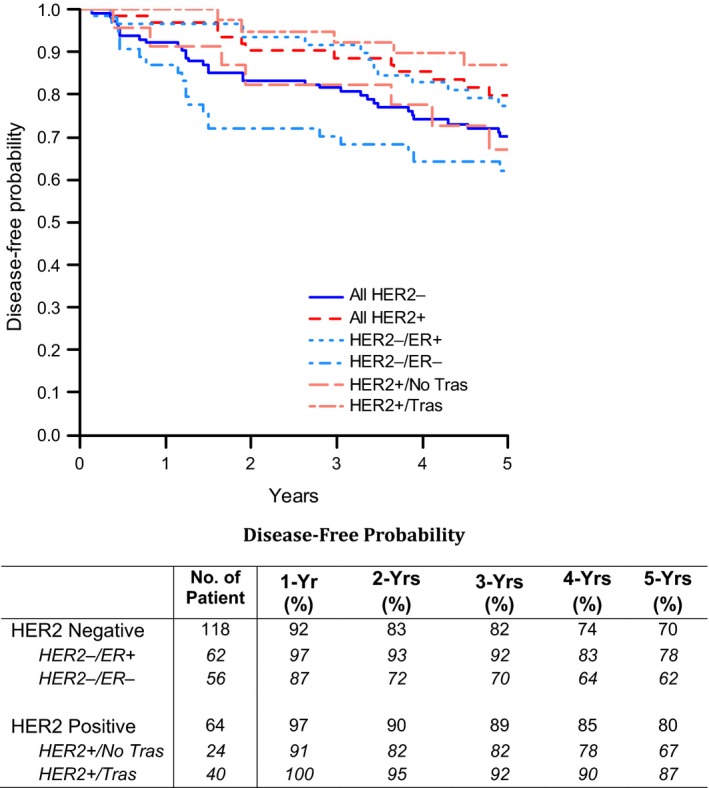
Disease‐free survival (ITT population).

**Figure 3:**
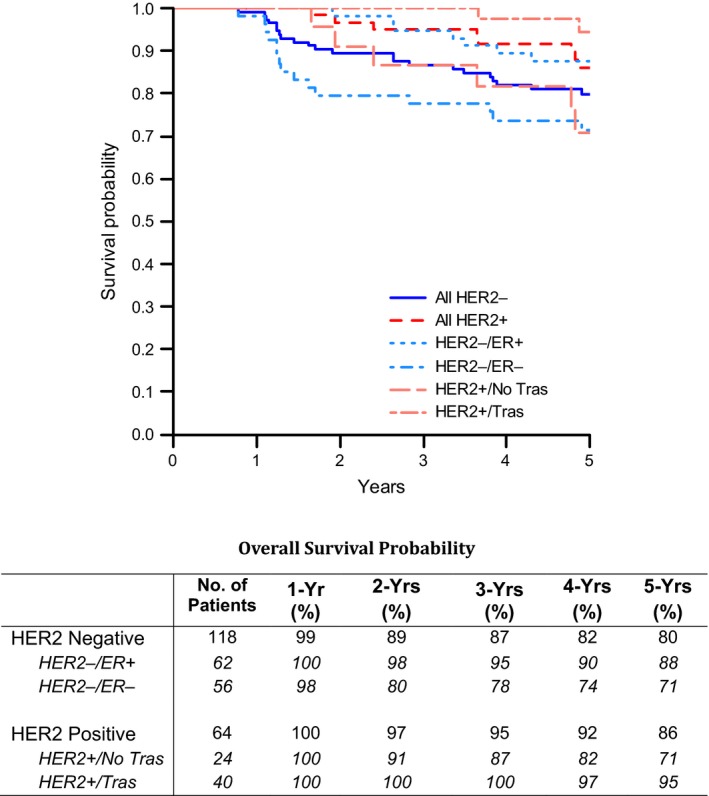
Overall survival (ITT population).

Five‐year DFS rates for the HER2‐negative and the trastuzumab‐treated HER2‐positive patients, respectively, were 70% (95% CI: 61–78%) and 87% (95% CI: 71–94%) (Fig. 2). 5‐year OS rates in the HER2‐negative and trastuzumab‐treated HER2‐positive patients, respectively, were 80% (95% CI: 71–86%) and 95% (95% CI: 80–99%) (Fig. 3). The 5‐year DFS and OS rates for the HER2‐negative and HER2‐positive patients who were not treated with trastuzumab, combined (*n* = 146), were 70% and 79%, respectively (data not shown). The 5‐year DFS and OS rates in the HER2‐negative patients based on ER status are presented in Figures 2 and 3.

### Toxicity

Grade 3 and 4 adverse events observed in the no trastuzumab and trastuzumab‐treated cohorts, respectively, were neutropenia: 83% and 68%; leukopenia: 37% and 25%; diarrhea: 10% and 8%; and hand‐foot skin reactions (grade 3 only): 10% and 8%. Of the 40 trastuzumab‐treated patients, five developed cardiac toxicity (2 grade 1; 2 grade 2; 1 grade 3), and all five patients’ LVEFs returned to normal upon completion of trastuzumab treatment. Table 3 summarizes treatment‐related Grade 3 and 4 adverse events.

**Table 3:** Treatment‐related Grades 3 and 4 adverse events (% patients)

Adverse event	No Trastuzumab		Trastuzumab^a^	
	Grade 3 (%)	Grade 4 (%)	Grade 3 (%)	Grade 4 (%)
Neutropenia	13.8	69.0	22.5	45.0
Leukopenia	26.2	11.0	22.5	2.5
Diarrhea	9.0	1.4	5.0	2.5
Hand‐foot skin reaction	9.7	0	7.5	0
Asthenia	6.9	0	5.0	0
Thrombocytopenia	4.8	2.1	0	0

a Patients treated with trastuzumab received 75 mg/m^2^ of epirubicin instead of 100 mg/m^2^ in the “No trastuzumab” group.

In the overall safety population, the median doses delivered per cycle were as follows: 5‐fluorouracil 500 mg/m^2^, docetaxel 70 mg/m^2^, cyclophosphamide 500 mg/m^2^, and 799 mg/m^2^ bid for capecitabine. The median delivered doses of epirubicin in the HER2‐negative and HER2‐positive patients, respectively, were 100 mg/m^2^ and 75 mg/m^2^. Dose modifications in patients treated without or with trastuzumab, respectively, included dose delays in 77 (53%) and 24 (60%) patients, dose reductions in 90 (62%) and 29 (73%) patients, and treatment discontinuations in 19 (13%) and three (8%) patients.

## Discussion

Our results showed that although well tolerated, preoperative FEC100 (or FEC75‐H) followed by wTX (or wTX‐H) was not a major improvement over currently available neoadjuvant chemotherapy regimens. The pCR rate of 31% in the HER2‐negative patient cohort is not substantially different than the reference pCR rate of 26% observed with preoperative AC followed by docetaxel in the NSABP B‐27 trial 1.

Our findings are consistent with results from other clinical trials that evaluated capecitabine as part of treatment regimens for patients with early‐stage breast cancer. In the adjuvant setting, the USON 01‐062 trial compared four cycles of doxorubicin/cyclophosphamide (AC) followed by four cycles of docetaxel every 3 weeks with or without capecitabine 875 mg/m^2^ BID days 1 thru 14 every 21 days, and found no difference in 5‐year DFS rates between treatments (HR = 0.84; *P* = .125), although 5‐year OS favored the capecitabine arm (HR = 0.68; *P* = .011) 20. However, in an exploratory analysis of centrally determined Ki67, O'Shaughnessy et al. 7 showed that for patients with Ki67 of <10% and <20%, only 2% and 6%, respectively of the 7 year disease free events occurred in these groups suggesting these patients with low proliferative tumors may not be informative for evaluation of chemotherapy trials. In the FinXX trial, patients with node‐positive or high‐risk node‐negative breast cancer were randomized to receive postoperative therapy with either three cycles of docetaxel/capecitabine followed by three cycles of cyclophosphamide/epirubicin/capecitabine or three cycles of docetaxel followed by three cycles of cyclophosphamide/epirubicin/fluorouracil. Although there was a trend toward improvement in 5‐year relapse‐free survival (RFS) (HR = 0.79; *P* = .087) and OS (HR = 0.73; *P* = .08), neither endpoint reached statistical significance 21. In elderly patients, the CALGB 49907 trial compared standard adjuvant therapy with either cyclophosphamide/methotrexate/fluorouracil (CMF) or AC versus capecitabine monotherapy and found that capecitabine had inferior efficacy with regard to both 3‐year RFS and OS 22. Similarly in the ICE trial, also in a population of elderly patients with breast cancer, no advantage in either 5‐year DFS or OS was observed with the addition of capecitabine to adjuvant therapy with ibandronate alone 23.

In the preoperative setting results from trials that have incorporated capecitabine have been mixed. In the ABCSG‐24 trial, the addition of capecitabine to epirubicin plus docetaxel significantly increased pCR rates 24. In the GeparTrio trial which evaluated the utility of response‐guided therapy, early non‐responders following two cycles of neoadjuvant therapy with docetaxel/doxorubicin/cyclophosphamide (TAC) were randomized to receive four additional cycles of TAC or four cycles of vinorelbine plus capecitabine (NX). There was no difference in pCR rates or OS on the two nonresponder study arms, although DFS was significantly improved with TAC‐NX compared with six cycles of TAC in the nonresponders, suggesting that pCR rates may not always accurately predict for systemic recurrence and survival endpoints 25, 26. In contrast, in the Gepar–Quattro trial, incorporating preoperative capecitabine either concurrently or sequentially with docetaxel following epirubicin/cyclophosphamide failed to improve pCR rates, DFS or OS as compared with docetaxel alone 27, 28. Likewise, in the NSABP B‐40 trial, adding preoperative capecitabine to docetaxel prior to AC had no significant impact on the pCR rate compared with docetaxel followed by AC 29. Ohno et al. 30 treated 504 stage I–III (T1 > 1 cm and N1) patients with FEC100 × 4 then either XT or docetaxel alone 100 mg/m^2^ per O'Shaughnessy et al. 6 and made five important observations. First, overall, there were no significant differences in pCR, disease‐free survival (DFS) or overall survival, between XT or docetaxel alone. However, and second, patients with a higher pretreatment Ki67 labeling index, in the midrange of 10–20%, compared to those with Ki67 < 10%, did show a trend towards a higher pCR with XT compared to docetaxel alone. Third, pretreatment KI67 labeling index was significant predictor of pCR. Fourth, pCR and posttreatment Ki67 labeling index <10% were significantly associated with longer DFS. Fifth, pretreatment Ki67 labeling index may be a useful biomarker to identify patients most likely to benefit from more intensive preoperative chemotherapy. These observations are consistent with present understanding of the biology underlying chemotherapy responses in more highly proliferative tumors and are an important component of the basis for multiple genomic predictors 31, 32.

In the HER2‐positive patients, we observed an increase in the pCR rate from 35% in the subset of patients treated with chemotherapy alone to 53% in those treated with chemotherapy plus trastuzumab. This finding is consistent with results from the randomized study by Buzdar et al. which demonstrated a significant increase in the pCR rate in HER2‐positive patients when trastuzumab was added to a standard preoperative chemotherapy regimen (65% vs. 26%; *P* = .016) 8. A subsequent update, which included 22 additional patients assigned the same trastuzumab regimen, had a pCR of 54.5% (32.2–75.6), identical to this study 33. Estimated 3‐year recurrence‐free survival was 100% in the trastuzumab‐treated cohort, versus 85% in the cohort treated with chemotherapy alone (*P* = .041) 33. Similarly, in the NOAH trial, patients with HER2‐positive breast cancer randomized to preoperative therapy achieved a significantly higher pCR rate than those who received chemotherapy alone (43% vs. 22%; *P* = .0007), and this was associated with improved 5‐year event‐free survival (58% vs. 43%; HR = 0.64; *P* = .016) 34, 35. Thus, the observed pCR rate of 53% and 5‐year DFS rate of 78% in the trastuzumab‐treated cohort in our study corroborate the results of the Buzdar et al. and NOAH trials and confirm substantial clinical benefit for HER2‐positive patients when trastuzumab is incorporated into preoperative chemotherapy regimens. However, the addition of capecitabine to FEC/wT plus trastuzumab does not appear to improve the pCR or 5‐year DFS rates achieved with anthracycline/taxane/trastuzumab preoperative regimens. Furthermore, Sparano subsequently showed weekly docetaxel was inferior to three‐weekly docetaxel [36] and Buzdar et al. later found no improvement in pCR rates when trastuzumab was administered concurrently with weekly paclitaxel then FEC75 compared to FEC75 alone followed by concurrent paclitaxel with trastuzumab 37.

In summary, the administration of preoperative therapy with FEC100 (or FEC75‐H) followed by wTX (or wTX‐H) was feasible and was associated with an acceptable safety profile. However, the inclusion of preoperative capecitabine did not add to the efficacy of FEC/docetaxel, with or without trastuzumab, and is therefore not supported for routine clinical use.

## Conflict of Interest

FAH presently has no conflicting interests. In 2011 and 2012, she was on the Speaker's Bureau for Roche–Genentech for pertuzumab and trastuzumab emtansine.
